# Mini-Invasive, Ultrasound Guided Repair of the Achilles Tendon Rupture—A Pilot Study

**DOI:** 10.3390/jcm10112370

**Published:** 2021-05-28

**Authors:** Łukasz Paczesny, Jan Zabrzyński, Marcin Domżalski, Maciej Gagat, Miron Termanowski, Dawid Szwedowski, Łukasz Łapaj, Jacek Kruczyński

**Affiliations:** 1 Orvit Clinic, Citomed Healthcare Center, 87-100 Torun, Poland; drpaczesny@gmail.com (Ł.P.); miron.termanowski@onet.eu (M.T.); dszwedow@yahoo.com (D.S.); 2 Department of General Orthopaedics, Musculoskeletal Oncology and Trauma Surgery, University of Medical Sciences, 61-701 Poznan, Poland; esperal@o2.pl (Ł.Ł.); jacek@man.poznan.pl (J.K.); 3 Orthopedic and Trauma Department Medical, Veteran’s Memorial Hospital, University of Lodz, 90-137 Lodz, Poland; marcindomzalski@yahoo.com; 4 Department of Histology and Embryology, Faculty of Medicine, Nicolaus Copernicus University in Torun, Collegium Medicum in Bydgoszcz, 87-100 Torun, Poland; m.gagat@cm.umk.pl

**Keywords:** Achilles tendon, sural nerve, percutaneous repair, ultrasound guidance

## Abstract

Percutaneous acute Achilles tendon rupture suturing has become a leading treatment option in recent years. A common complication after this mini-invasive procedure is sural nerve injury, which can reduce the patients’ satisfaction and final outcomes. High-resolution ultrasound is a reliable method for localizing the sural nerve, and it can be performed intra-operatively; however, the long-term results are yet unknown. The aim of the study was to retrospectively evaluate the long-term results of percutaneous Achilles tendon repair supported with real-time ultrasound imaging. We conducted 57 percutaneous sutures of acute Achilles tendon rupture between 2005 and 2015; 30 were sutured under sonographic guidance, while 27 were performed without sonographic assistance. The inclusion criteria were acute (less than 7 days) full tendon rupture, treatment with the percutaneous technique, age between 18 and 65 years, and a body mass index (BMI) below 35. The operative procedure was carried out by two surgeons, according to the surgical technique reported by Maffulli et al. In total, 35 patients were available for this retrospective assessment; 20 (16 men and 4 women) were treated with sonographic guidance, while 15 (12 men and 3 women) underwent the procedure without it. The mean follow-up was 8 years (range, 3–13 years). The sural nerve was localized 10 mm to 20 mm (mean, 15.8; SD, 3.02) laterally from the scar of the Achilles tendon tear. There was no significant difference between groups with respect to the FAOQ score (*P* < 0.05). High-resolution ultrasounds performed intra-operatively can minimize the risk of sural nerve injury during percutaneous Achilles tendon repair.

## 1. Introduction

The Achilles tendon (AT) is the strongest and largest tendon in the body [1]. This large structure can bear 12.5 times the weight of the body during jogging [2]. Ultrasonography effectively represents histopathological changes in the AT, and, especially in Europe, it is regarded as the primary imaging method [3]. Achilles tendinopathy with degenerative alterations occur in 5.9% of seniors and 50% of elite athletes [4]. Degenerative tendinopathy is the most common histological finding in spontaneous tendon ruptures. Tendon degeneration may lead to reduced tensile strength and a predisposition to rupture [3]. The Achilles tendon is especially prone to injury among patients who are exposed to risk factors. These factors can be classified as extrinsic (i.e., training errors during sports activities or using the wrong equipment) or intrinsic. Intrinsic factors can be linked to biomechanics, such as increased foot pronation, a tight gastrocsoleus complex, cavus foot, or specific medical conditions, e.g., endocrine diseases, oral contraceptives, obesity, hormone replacement therapy, steroids, or fluoroquinolone antibiotic therapy [5,6,7]. The acute Achilles tendon rupture rate is estimated at 11 to 37 per 100,000 and is increasing [8]. The majority of tears are related to sports activities, with 53% attributable to jogging- and jumping-related sports (volleyball, basketball, and badminton) [9].

There are various Achilles tendon rupture treatment methods, including operative treatment (open, percutaneous, or mini-invasive repair) and non-operative treatment (cast, brace, or orthosis). No consensus has been reached regarding a universal treatment option. Surgical treatment always carries a risk of infection, as well as of wound healing complications, adhesions, and iatrogenic nerve injury [10].

The determination of treatment procedure was influenced by studies on the special role of the paratenon in Achilles tendon regeneration. It is crucial not to damage the vascularization during treatment [11]. In recent years, the protocols of surgical treatment have shifted from open to mini-invasive repairs [12]. When following those procedures, the structure of the paratenon is less damaged. Both open and percutaneous surgery always entails the risk of damaging the nerves and causing wound-related complications [8]. Van Maele et al. reported a complication rate of 40% after open repair, including sural nerve hypoesthesia (14.3%), delayed wound healing (28.6%), infection (20.9%), and re-rupture (4.8%) [13]. The development of minimally invasive surgery of the AT allowed a reduction in the wound complications rate. However, percutaneous techniques increase the risk of sural nerve (SN) entrapment, with the chance of SN neuritis ranging from 1.7% to 27% [10,14,15,16,17,18,19,20,21]. Some special instruments have been developed that reduce the risk of sural nerve entrapment, but these also have substantial disadvantages, i.e., the extra cost and additional learning curve [22,23]. SN entrapment could be extremely uncomfortable, thus reducing patient satisfaction. Usually, it causes neuropathic, burning pain, and paresthesia in the posterior and lateral aspects of the ankle and the foot [24]. Ultrasound (US) has been proven as a reliable method for assessing sural nerve development and potential entrapment [25]. After sterile draping of the US probe, it could easily be used intraoperatively. Giannetti et al. combined ultrasound and percutaneous Achilles surgery. They reported excellent results with no SN complications at 13-month follow-up [26].

The aim of the study was to retrospectively evaluate the long-term results of percutaneous Achilles tendon repair supported with real-time ultrasound imaging.

## 2. Materials and Methods

### 2.1. Patients

All patients admitted to the Department of Orthopedics for Adults and Children of Provincial Polyclinical Hospital and Orthopedic Department of Cito Care Hospital between 2005 and 2015 with Achilles tendon rupture were retrospectively reviewed. The inclusion criteria were acute (less than 7 days) full tendon rupture, treatment with percutaneous technique, age between 18 and 65 years, and a body mass index (BMI) below 35. 

Patients with a history of surgical treatment of the Achilles tendon were excluded from the study. 

### 2.2. Methods 

There was no randomization or special patient selection. Patients were treated at one of the two Orthopedic centers, depending on their admission; however, US was only available in one of these facilities. US was not used for sural nerve visualization during surgery at the Provincial Policlinic Hospital but was used as a standard at the Cito Care Hospital. All procedures were performed by two surgeons (LP and MT).

The retrospective assessment included the analysis of patient history, with a focus on Achilles re-ruptures, infections, and sural nerve-related complaints. Any complaints of pain and hypoesthesia of the external border of the foot were registered as sural nerve lesion. Ultrasound assessments of Achilles tendon structure and sural nerve position were performed with measurement of the distance between the border of the Achilles scar and the sural nerve at the level of the tear. The probe was localized perpendicular to the long axis of the limb, in the axial plane. Any abnormalities in scar formation, i.e., fluid reservoirs or poor stump approximation, were also registered.

During clinical examination, the ankle range of motion (ROM) and sensation deficit were analyzed. The Foot and Ankle Outcomes Questionnaire (FAOQ) with Polish cultural adaptations validated by Boszczyk et al. [27] was utilized to report the subjective outcome.

### 2.3. Surgical Procedure

**Figure 1:**
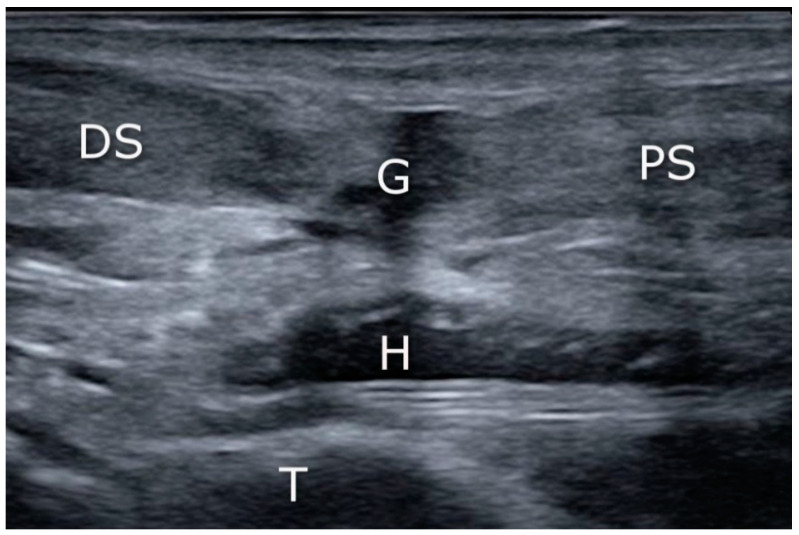
Achilles tendon tear: the sonographic appearance of acute Achilles tendon tear in the sagittal plane. G: gap (tendon lesion); H: hematoma; PS: proximal stump; DS: distal stump; T: tibia.

**Figure 2:**
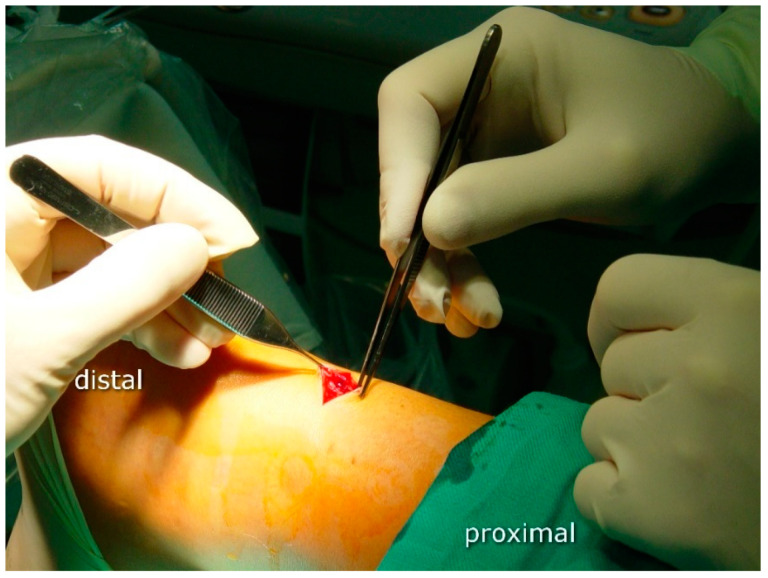
Skin cut: the first stage of surgery on the right Achilles tendon; the skin is transversely cut to make an approximately 2 cm incision directly over the tendon’s lesion, localized by means of US.

The operative procedure was performed by two surgeons with considerable experience in minimally invasive orthopedic surgery and musculoskeletal ultrasound. The surgical procedure was performed under regional or general anesthesia with a calf tourniquet. The patient was positioned prone on the operating table. The skin was prepared, and the sterile drape was used in the standard fashion. In the US-assisted group, the first step was to visualize the site of the rupture by means of ultrasound (linear probe 10–12 Mhz) (Figure 1). In the non-guided group, the rupture site was located by palpation. A transverse incision was made over the Achilles tendon defect using a surgical blade (size 11), and the tissue was spread with forceps to expose the paratenon (Figure 2). 

**Figure 3:**
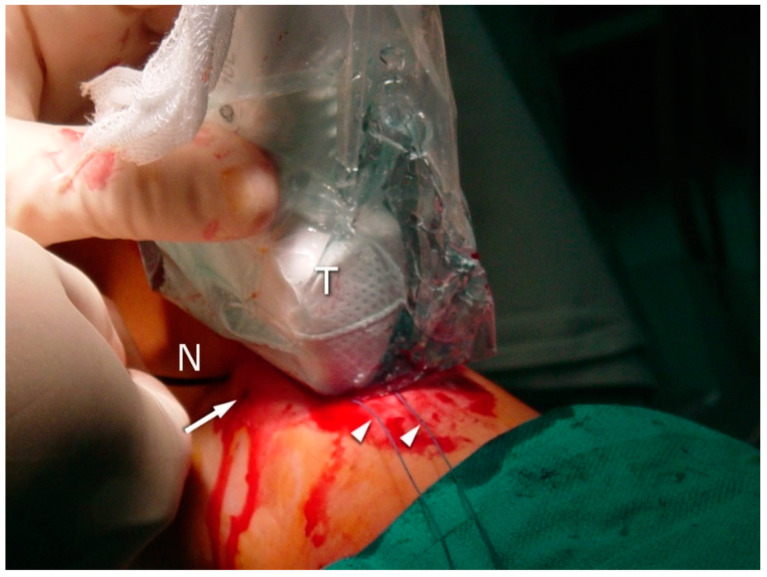
The needle: the second stage of the surgery; distal sutures are performed by placing the needle (N) distally under the transducer (T) control. Note the puncture (arrow) resulting from the first suture (arrowhead).

**Figure 4:**
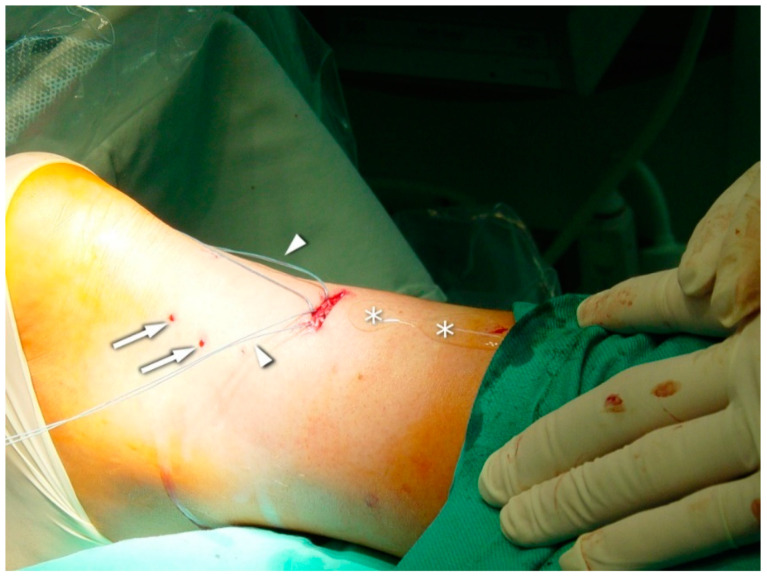
Distal sutures: the beginning of the third stage; note the punctures (arrows) resulting from distal suture deployment (arrowheads). Ultrasonic gel (asterix (*)) facilitates visualization during the placement of the proximal sutures.

**Figure 5:**
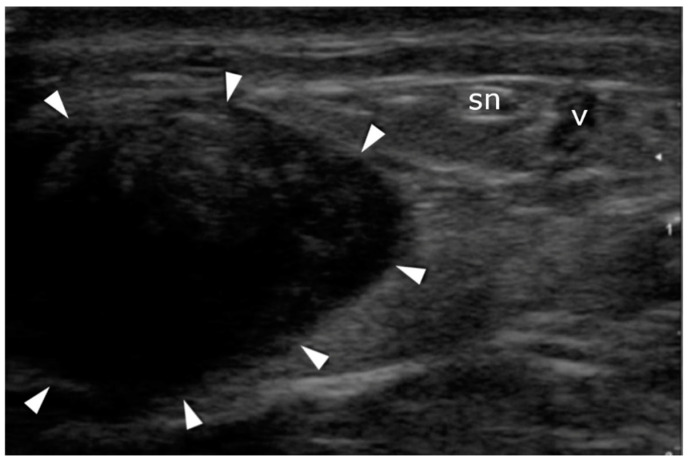
Sural nerve identification: transverse plane. The nerve (sn) and vein (v) pass on the lateral side of the Achilles tendon (arrowheads).

Six sutures (Number 1 Maxon), three of which were situated proximally and the other three distally (2, 4, and 6 cm) to the rupture side, were passed transversally through the tendon stumps. In the US-guided group, sonographic visualization was performed as discussed below to pass the needle through the center of the tendon and avoid the sural nerve (Figure 3, Figure 4 and Figure 5). In the non-guided group, the surgeons relied on characteristic anatomical spots and tissue palpation during suture deployment. In both groups, forceps were used to separate the AT from the subcutaneous tissue. The sutures were then passed back out to the area of the initial transverse incision. Plantar flexion of 20° was applied. The sutures were tied to allow stump approximation and buried in the subcutaneous layer of the tissue. The skin incisions were closed using non-absorbable sutures (Number 3.0 Dafilon) and sterile dressings were applied. The extremity was immobilized in a below-knee walker brace with heel support to maintain 20° plantar flexion. 

### 2.4. Ultrasound Imaging in the US-Guided Group

**Figure 6:**
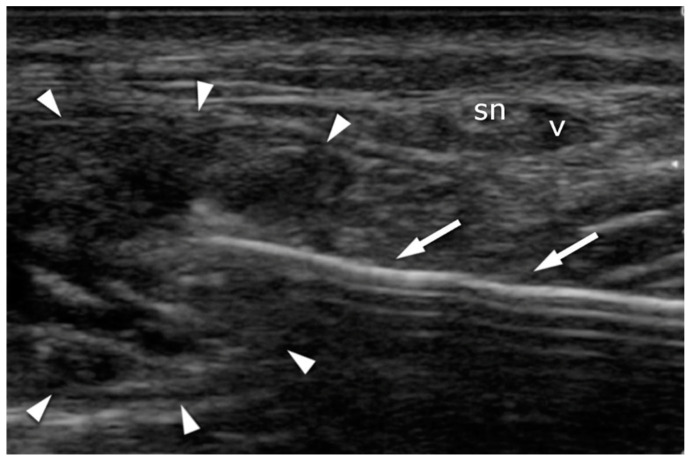
Achilles tendon (arrowheads) suturing: transverse plane. The needle (arrows) is introduced under US guidance in order to avoid contact with the sural nerve (sn) and the vein (v).

**Figure 7:**
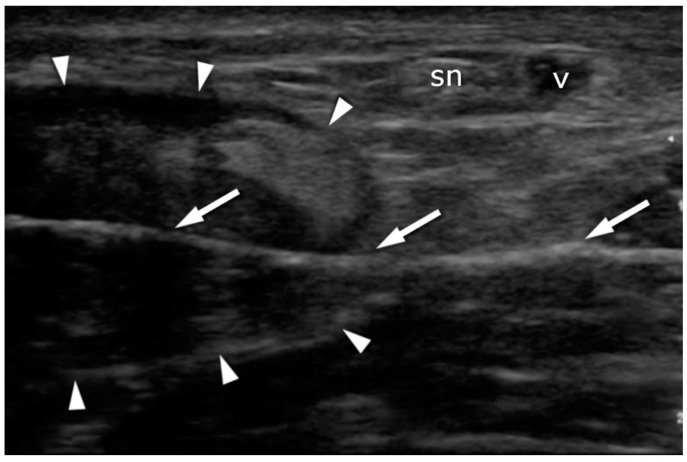
Achilles tendon (arrowheads) after suture deployment: transverse plane. Note that the suture placement (arrows) avoids contact with the sural nerve (sn) and the vein (v).

A high-resolution real-time ultrasound examination was performed in the operation room. We used a sterile draped high-frequency (10–12 MHz) linear probe. Transverse scans were used to localize the SN. It was followed from the proximal area of the calf—where it laterally follows the saphenous vein in its fascial compartment between the two heads of the gastrocnemius—to the distal area and the lateral malleolus area. The SN’s echogenicity is oval and typical of nerves, showing hyperechogenic spots in the hypoechogenic area surrounded by the hyperechogenic sheath (Figure 5, Supplementary Material Video S1). The entry point and the angle of the needle were chosen with direct US guidance (Figure 6, Supplementary Material Video S2). The suture course was checked before it entered the skin incision (Figure 7, Supplementary Material Video S3).

### 2.5. Aftercare

All patients were advised to follow the same postoperative protocol. Initially, they were immobilized in a walker brace and their plantar flexion was set at 20 degrees. Patients were allowed to walk with crutches with no weight bearing for 2 weeks and then could progress with the weight bearing as tolerated. Early clinical examination was performed 10–14 days after the surgical procedure, and all patients underwent wound inspection with US assessment of the approximated AT stumps. The sutures were removed, and at this stage, physiotherapy (PT) was commenced as advised, with brace removal during the PT sessions and controlled movement supervised by the physiotherapist. The brace position had to be gradually adjusted toward dorsal flexion depending on the patient’s tolerance. Six weeks after surgery, another US inspection of the AT and an assessment of the range of motion in the ankle joint were performed. The brace was completely removed at this stage, after which the PT protocol was continued. 

### 2.6. Statistical Analyses

Data were tested for normality by means of histograms. The Mann–Whitney U test was used to assess the statistical significance of the FAQQ score, and Fisher’s exact test was used to assess complications (Social Science Statistics, 2018).

## 3. Results

In total, we identified 57 patients who had undergone percutaneous repair of acute Achilles tendon rupture; 30 of these were sutured under US guidance, and 27 without sonographic assistance. After 5 years, contact was lost with 22 subjects, but 35 were available for the control examination. Of these, 20 (16 men and 4 women) were treated under US guidance, while 15 (12 men and 3 women) underwent the procedure without it. The mean follow-up was 8 years (range 3–13 years). A comparison between the groups can be found in Table 1.

### 3.1. FAOQ

There was no significant difference between groups with respect to the FAOQ score (Mann–Whitney U = 124.5, *P* < 0.05 two-tailed). 

### 3.2. Re-Ruptures

Two re-ruptures of the tendon in the US-assisted group occurred two months after the primary surgery, and these were treated with the open procedure. Both patients had not followed the PT protocol. The first patient was immobilized for 6 weeks. The second patient removed the boot after 4 weeks and recommenced full weight-bearing exercises; the rupture occurred during a dynamic split squat. The injury became infected after a secondary suture and required a third procedure. It healed well, and the patient returned to recreational sports activities. There were no re-ruptures in the non-US group. The difference between groups in terms of re-ruptures and infections was insignificant (the Fisher’s exact test statistical value for re-ruptures was 0.4958, and 1 for infections; *p* < 0.01).

### 3.3. Sural Nerve Complications

No persistent sural nerve-related complaints were reported in the US-guided group. The non-guided group experienced a high rate of SN dysfunction—3 of 15 cases. All three patients reported numbness and mild paraesthesia in the lateral calf and heel. These sensations started directly after the surgery and were still present at the follow-up. The Fisher’s exact test statistical value for group difference was 0.0695. The *p*-value was between 0.05 and 0.1—a notable trend approaching close to significance. 

### 3.4. US Achilles Assessment 

At the final follow-up, Achilles tendon healing was confirmed in all patients with hyperechoic scarring and appropriate stump approximation. Both re-ruptured tendons also showed signs of adequate healing after open revision surgery. 

### 3.5. US Sural Nerve Assessment 

The SN was identified in all subjects. The SN was localized 10 to 20 mm (mean 15.8, SD 3.02) laterally from the scar at the tear level. No sutures around the sural nerve, or any discontinuity/lesions of the nerve, were observed in any of the three patients complaining of persistent paresthesia.

## 4. Discussion

We received no sural nerve-related complaints in the US-guided group, but re-rupture and infection only occurred after the use of this technique. 

These results should be treated with caution, as there are many limitations to our study. The most important is its retrospective design, with no randomization. Relatively small groups of patients were assessed after a long follow-up. Due to such a long follow-up, we lost contact with a substantial number (22/57) of our patients. Statistical analyses performed on such small samples are weak. Another limitation is the lack of some important patient data, i.e., comorbidities, body mass index, or associated injuries, which could have biased the results.

Webb and Bannister described a technique of performing three sutures in line with the AT to avoid iatrogenic SN damage [28]. Their technique required three horizontal skin incisions. They observed no SN injuries or re-ruptures in their 27 subjects but reported one wound abscess and one case of complex regional pain syndrome. Due to the small groups in both their study and our own, it is impossible to state if those complications were related to the different number of skin incisions, the different suture deployment techniques, or other factors. Cretnik et al. conducted a comparative study of 132 patients treated with percutaneous and open-surgery techniques [29]. The complication rate was over two times lower for minimally invasive surgery, but SN injury occurred in 4.5% of subjects, and the re-rupture rate was 3.7%. The long-term results of the larger cohort were also analyzed by Cretnik et al. They determined rates of 2.96% re-ruptures and 5.18% transient sural nerve neuropathies [30]. Carmont and Maffulli presented a percutaneous technique that was predicted to further reduce SN trauma [15]. They stated that even if nerve injury does occur as a result of their technique, it is more likely to be a longitudinal neurotomy rather than a transverse cut. Majewski et al. compared two techniques—one with exposure of the sural nerve and the other without—and noticed sural nerve-related complications in 18% of patients in the non-exposure group [16]. 

Tenolig (FH Orthopedics, Heimsbrunn, France) is a device developed to facilitate Achilles tendon repair. Although the risk of infection after this procedure is low, Maes [31] reported re-rupture in 10% of cases and sural nerve injury in 6.4%. Lacoste et al. used US to visualize the SN during Tenolig repair. Using the same guidance technique as we did, they reduced sural nerve complication to 2.6%. All of these were transient and resolved spontaneously within 4 months. No patient in their cohort experienced permanent sural nerve damage or deep infection [32].

The technique described in our study allows for more versatile suture placement than can be achieved with special techniques, such as those using Tenolig or Achillon. The use of a free-hand straight needle with US visualization allows for reliable suture deployment. The direction and angle of the needle can be selected as required to avoid SN. No signs of sonographic discontinuity or sutures around the nerve were found in our patients with persistent sural nerve symptoms. The nerve may have been simply punctured/injured during the surgery, leaving persistent dysfunction but no US signs at the long follow-up.

There are few reports on the use of intraoperative US during Achilles repair. The first is the work of Blankstein et al. They described the use of US guidance for better stump approximation [33]. In 2010, Soubeyrand et al. published a study in which they proved that 45% of needles placed without US guidance require repositioning. They used US to achieve this, with satisfactory results [34]. They concluded that intraoperative US is useful for both the confirmation of needle position and for stump approximation. None of these authors used US to visualize the SN during the procedure, as we did. 

In 2014, Giannetti et al. reported a prospective use of US during percutaneous surgery. As in our study, they did not note SN injury at their 13-month follow-up [26]. Suture placement was carried out via the original Ma and Griffith technique, with each suture passing the proximal stump transversely and then obliquely, in the Bunnel fashion. We only used transverse sutures, placed perpendicular to the Achilles tendon with no oblique passes. It is unclear whether this fact caused the high re-rupture rate (2/20; 10%) in our US-guided group. It should be noted that both re-ruptures occurred in patients that failed to comply with the postoperative protocol. Presumably, using larger groups would increase our knowledge of the risk factors of this severe complication.

In 2019, in their technical note, Severyns et al. described the ultrasonographic visualization of the ruptured tendon for sural nerve visualization before endoscopic portal placement during endoscopic Achilles tendon repair [35]. Their idea was similar to ours—to minimize the risk of SN injury during Achilles repair—but they used it during the endoscopic repair. So-called double-assisted surgery (both ultrasonographically and endoscopically guided) could reduce the complication rates even further, but this requires comparative studies to confirm our technique in the future.

Real-time intra-operative US imaging of the SN requires experience and proper skills. It should be noted that intra-operative US prolongs the operative procedure and increases the cost. The potential applicability of US in reducing SN iatrogenic lesions and its possible impact on the occurrence of complications require further prospective assessment on larger groups of patients.

## 5. Conclusions

The intra-operative utilization of high-resolution ultrasound could minimize the risk of sural nerve injury during percutaneous Achilles tendon repair. 

## Figures and Tables

**Table 1:** Patients’ data.

No.		US Guided Group	Non-Guided Group
		20	15
Age (years)	Range	30–62	30–60
	Mean	42	43
	SD	8.95	9.98
Follow-up (years)	Range	3–13	5–10
	Mean	8	8
	SD	3.32	1.51
FAOQ score (pts)	Range	55–90	58–86
	Mean	78	76
	SD	9.65	8.35
Complications	Re-ruptures	2	
	SN lesions		3
	Infections	1	

## Data Availability

The data presented in this study are available on request from the corresponding author.

## Supplementary Materials

The following are available online at https://www.mdpi.com/article/10.3390/jcm10112370/s1, Video S1: Localization of the SN, Video S2: Introduction of the needle, Video S3: Visualization of the suture course.
